# Mental health-related sickness absences in parents of children with mental disorders or neurodevelopmental conditions

**DOI:** 10.1017/S2045796025100395

**Published:** 2025-12-19

**Authors:** Mai Gutvilig, Kaisla Komulainen, Ripsa Niemi, Laura Pulkki-Råback, Marko Elovainio, Christian Hakulinen

**Affiliations:** 1 Department of Psychology, Faculty of Medicine, University of Helsinki, Helsinki, Finland; 2 Finnish Institute for Health and Welfare, Helsinki, Finland

**Keywords:** adolescence, child psychiatry, economic issues, families, mental health

## Abstract

**Aims:**

Having a child with a psychiatric diagnosis is associated with parents’ greater risk of subsequent mental disorders but no immediate change in their annual labour market metrics. This discrepancy could be explained by shorter absences from work. We examined first-time psychiatric sickness absences in parents whose children have psychiatric diagnoses.

**Methods:**

Using several linked nationwide Finnish registers, in this cohort study we examined time to first psychiatric sickness absence in parents whose children were born in 2001–2012 (early-childhood-onset diagnoses) or 2005–2016 (late-childhood-onset diagnoses). Exposure was having a child with a psychiatric diagnosis. Follow-up started when the parent’s eldest turned 1 (early-childhood-onset diagnoses) or 5 (late-childhood-onset diagnoses) and ended at psychiatric sickness absence, emigration, 68th birthday, death, or 31 December 2020, whichever occurred first.

**Results:**

The 2001–2012 and 2005–2016 cohorts included 357 135 and 397 874 parents followed for 3.31 and 3.70 million person-years. Having a diagnosed child was associated with greater risk of psychiatric sickness absence in all except men whose children had substance use or psychotic disorder diagnoses. Time-varying analyses showed the greatest associations for women (HR: 4.92; 95% CI: 3.97–6.10) and men (HR: 2.48; 95% CI: 1.61–3.80) within 6 months of a child’s eating disorder diagnosis.

**Conclusions:**

Parents of children with psychiatric diagnoses may be at a greater risk of a psychiatric sickness absence. Associations differed by child’s diagnosis, parent’s gender and time since diagnosis.

## Introduction

A child’s mental disorder or neurodevelopmental condition does not only influence the child themselves but may also impact their family. Parenting a child with a mental disorder or neurodevelopmental condition often involves high care demands and may lead to coping difficulties and psychological distress (Cham *et al.*, 2022; Fox *et al.*, 2017; Kamil and Velligan, 2019; Phillips *et al.*, 2023). Consequently, parents have reported challenges in balancing caring for their child with paid employment (Brown and Clark, 2017; Sellmaier, 2019) potentially leading to a reduced ability to work. Recently, however, our two studies (Hakulinen *et al.*, 2025; Komulainen *et al.*, 2025) showed that while parenting a child with a mental disorder was indeed associated with greater risk of psychiatric morbidity, this did not translate into changes in labour market outcomes around the time of the child’s diagnosis. One potential explanation for this discrepancy could be that the parents’ psychiatric distress may necessitate shorter work absence spells that would not be observed in annual labour market metrics. These shorter absences could, for example, be sickness absences.

In Finland, an individual is eligible for a physician-certified sickness absence if a disorder impairs their ability to work and their condition would deteriorate without the sickness absence. Currently in Finland, the leading cause of sickness absence is a mental or behavioural disorder diagnosis of the International Statistical Classification of Diseases and Related Health Problems 10th Revision (ICD-10) (Kela, 2025b). These sickness absences, henceforth referred to as psychiatric sickness absences for brevity, carry both economic and social consequences. Namely, psychiatric sickness absences are a major risk factor for complete exclusion from the job market (Alexanderson *et al.*, 2012). Moreover, diminished work participation may reduce the social benefits that employment provides, such as helping to cope with other life stressors (Morris, 2012, 2014) thus potentially exacerbating the psychiatric distress individuals experience.

Only few studies have examined associations between parenting a child with a psychiatric diagnosis and parents’ sickness absences. A Norwegian study found that being a mother of a child with any severity of special healthcare needs was associated with a significantly greater risk of psychiatric sickness absence compared to mothers of children without special healthcare needs (Hauge *et al.*, 2015). However, as the study was on special healthcare needs overall, it also included children with somatic disorders while also only examining mothers of children aged 1–4 years. Two Swedish studies examined sickness absences in parents of children with autism spectrum disorder and schizophrenia (McEvilly *et al.*, 2015; Mittendorfer-Rutz *et al.*, 2019). Both found that having a diagnosed child was associated with a greater risk of sickness absences relative to other parents but only one analysed mothers and fathers separately finding the associations stronger in mothers compared to fathers.

The aim of this study was to comprehensively examine associations between children’s psychiatric diagnoses across ICD-10 subchapter F categories, which include all mental disorders and neurodevelopmental conditions, and parents’ first psychiatric sickness absences. Using Finnish nationwide registers, we provide estimates that are specific to the parent’s gender, child’s diagnosis, and time since the diagnosis. Additionally, we explored associations with short and long sickness absences separately.

## Methodology

### Study design and population

When investigating diagnoses with a typically early childhood onset (intellectual disabilities, developmental disorders and childhood onset disorders; ICD-10: F70–F98) we were mainly interested in parents during their children’s early childhood. Conversely, when examining disorders with a later onset (substance use, psychotic, mood, anxiety and eating disorders; ICD-10: F10–F50), we were predominantly interested in parents during their children’s adolescence. Data on parents’ sickness absences was available from 2003–2020 with 2003–2005 reserved for washout. If we were to follow parents of children born in 2006 the earliest, we could only follow families until the eldest children turned 14. This would exclude many late-childhood-onset diagnoses and redundantly follow families during their children’s early childhood when studying disorders only occurring later in development. Therefore, to maximise the use of data, we built two birth cohorts:

1. Early-childhood-onset conditions: All Finnish residents whose children were born in Finland in 2005–2016. We started following the parents when their eldest was deemed old enough to receive a diagnosis (1 year old) bringing the eldest children to 15 years old at the end of follow-up.
2. Late-childhood-onset conditions: All Finnish residents whose children were born in Finland in 2001–2012. We started following the parents when their eldest was five years old resulting in the eldest children being 19 at the end of follow-up.

Parents belonged to both birth cohorts if their children were all born in 2005–2012.

Data were compiled by linking Finnish registers using pseudonymised personal identification numbers allocated to each Finnish resident. Demographic characteristics and family links were retrieved from the full Finnish Population Register (FOLK) maintained by Statistics Finland, which contains yearly updated administrative registers for all permanent residents in Finland. Children and parents’ mental disorders were retrieved by linking physician-made diagnoses from multiple registers all maintained by the Finnish Institute for Health and Welfare: The Hospital Discharge Register (inpatient hospitalisations 1970–1993), and the Care Register for Health Care (inpatient 1994 onwards, outpatient 1998 onwards), which include diagnoses from public and private secondary care along with emergency department visits, and the Register of Primary Healthcare Visits (2011 onwards), which includes public and private visits to primary healthcare. Sickness absences were obtained from the sickness allowance register of the Social Insurance Institution of Finland (Kela), which includes all sickness absences certified by a physician from the 10th absence day forward. The cut-off is based on the Finnish sickness insurance system where Kela provides, and consequently records, earnings-tied sickness absence compensation from the 10th day of absence onward. The record includes the start and end of the sickness absence compensation period, and the diagnosis based on which it was certified. Finnish sickness absence compensation can be granted to any 16–67-year-old resident – regardless of employment status – who is not currently receiving any long-term compensation for reduced ability to work (e.g., rehabilitation subsidy or disability pension) (Kela, 2025a). Authors had full access to the database population used to create the study population. Details on the data cleaning process can be found in the Supplemental Methods.

We examined first-time psychiatric sickness absences in parents of children with a mental disorder or neurodevelopmental condition diagnosis compared to those whose children were undiagnosed at the time. Any parents who received a psychiatric sickness absence prior to the beginning of follow-up or were younger than 16 at the beginning of follow-up were excluded from the study population. Follow-up ended at the parent’s sickness absence, death, permanent emigration from Finland, 68th birthday, or 31 December 2020, whichever occurred first.

The Ethics Committee of the Finnish Institute for Health and Welfare approved the study plan (THL/184/6.02.01/2023§933). Data were linked with the permission of Statistics Finland (TK-53-1696-16), the Finnish Institute of Health and Welfare and the Social Insurance Institution of Finland.

## Measures

### Exposures

A child’s mental disorder was defined using the ICD-10 subchapter F categories disregarding organic mental disorders (F00–F09) and personality disorders (F60–F69) as few of these diagnoses occur before the age of 18. Additionally, to stay consistent with previous research (Hakulinen *et al.*, 2025; Komulainen *et al.*, 2025), we only analysed eating disorders (F50) from behavioural syndromes associated with physiological disturbances and physical factors (F50–F59) and pervasive developmental disorders (F84) from disorders of psychological development (F80–F89). Some primary healthcare facilities in Finland use the International Classification of Primary Care, 2nd edition (ICPC-2), in which case diagnoses were translated into ICD-10. For details on the diagnostic categories, see Supplemental Table S1.

The date of the child’s first diagnosis in each diagnostic category received under the age of 18 was considered the parent’s date of exposure. If one child had diagnoses from multiple diagnostic categories, parents were exposed in all those analyses. In the case of several children of the same parent receiving a diagnosis within the same category, the earliest date was the exposure.

### Outcomes

First psychiatric sickness absence was defined as the earliest date of any sickness absence associated with an ICD-10 subchapter F diagnosis. When short and long sickness absences were investigated separately, two events were defined: first short sickness absence (<8 weeks) and first long sickness absence (≥8 weeks) associated with an ICD-10 subchapter F diagnosis.

### Confounders

We included the following time invariant variables as potential confounders: the year follow-up started, parent’s gender (female/male as registered in February, 2025), parent’s age at the birth of their first child (1 = below 20, 2 = 20–24, 3 = 25–29, 4 = 30–34, 5 = 35–39, 6 = 40–44, 7 = ≥ 45), and parent’s education at the beginning of follow-up (1 = primary, 2 = upper secondary, 3 = higher education). Moreover, we included the following time-varying variables: parent’s psychiatric history prior to exposure (0 = no ICD-10 subchapter F diagnoses (or corresponding ICD-8/ICD-9 diagnoses), 1 = ≥1 diagnoses), and eight variables measuring comorbidities in the family: Seven of these variables indicated whether the parent’s children had a diagnosis in each of the diagnostic groups, except the exposure at hand, prior to the exposure (0 = no child of the parent had a diagnosis in the category, 1 = ≥1 diagnoses in the category), and one count variable indicated how many diagnostic categories, other than the exposure at hand, the parent’s children had a diagnosis in prior to the exposure (0 = 0 or 1 diagnostic category, 2 = 2 diagnostic categories, 3 = 3 diagnostic categories, 4 = ≥4 diagnostic categories).

### Statistical analysis

We used Cox proportional hazards models to examine time to a parents’ first psychiatric sickness absence relative to having a child with a psychiatric diagnosis. Exposure was time-varying, i.e., each parent was unexposed until the date of their child’s diagnosis and exposed thereafter. Gender-specific estimates were obtained with an interaction term between parent’s gender and the exposure. Children’s diagnostic categories were analysed separately. Next, we relaxed the proportional hazards assumption by analysing five time periods after exposure (0–6 months, >6–12 months, >1–1.5 years, >1.5–2 years and >2 years). When hazards were not proportional, they were interpreted as the average across the time period (Stensrud and Hernán, 2020). Finally, we examined time to a short and long sickness absence as distinct outcomes where the other outcome was a competing risk. We handled competing risks by computing cause-specific hazard ratios, where the alternative outcome was censored (Schuster *et al.*, 2020). All analyses were adjusted for the aforementioned confounders. Individuals with missing information on education were excluded from analyses (2001–2012 cohort = 385; 2005–2016 cohort = 1852).

Data management and analyses were done in Stata version 17.0 (StataCorp). Figures and tables were done in R version 4.2.2 (R Core Team, 2023) using packages tidyverse (Wickham *et al.*, 2019), ggpubr (Kassambar, 2023), ggsignif (Ahlmann-Eltze and Patil, 2021) and gtsummary (Sjoberg *et al.*, 2021).

## Results

The 2005–2016 cohort consisted of 198 064 women and 199 810 men who were followed for 1.82 and 1.88 million person-years, respectively. The 2001–2012 cohort included 177 890 women and 179 245 men who were followed for 1.62 and 1.69 million person-years. Table 1 presents the baseline characteristics of the study population. Details on the number of parents excluded due to prior psychiatric sickness absences, new absences during follow-up, parents censored due to alternative outcomes and exposed parents can be found in Table 2. The most common reasons for parents’ sickness absences were anxiety, depressive and sleep disorders (for details, see Supplemental Table S2).

**Table 1.** Demographic characteristics of the study populations

Characteristic		Women		Men	
		2001−2012 Cohort	2005−2016 Cohort	2001−2012 Cohort	2005−2016 Cohort
		Median (IQR)	Median (IQR)	Median (IQR)	Median (IQR)
Parent’s birth year, *N* (%)					
	<1960	650 (0.4%)	51 (<0.1%)	4109 (2.3%)	1549 (0.8%)
	1961−1970	28125 (16%)	10125 (5.1%)	42744 (24%)	20528 (10%)
	1971−1980	109068 (61%)	92325 (47%)	104570 (58%)	102983 (52%)
	1981−1990	38972 (22%)	87095 (44%)	27235 (15%)	69781 (35%)
	>1990	1075 (0.6%)	8468 (4.3%)	587 (0.3%)	4969 (2.5%)
Parent’s age at start of follow-up		34 (30, 37)	30 (26, 33)	35 (32, 39)	31 (28, 35)
Parent’s age at end of follow-up		43 (39, 47)	39 (35, 43)	45 (41, 49)	41 (37, 45)
Parent’s education at start of follow-up, *N* (%)					
	Lower secondary or less	18701 (11%)	28222 (14%)	27261 (15%)	35600 (18%)
	Upper secondary	63031 (35%)	72815 (37%)	83217 (46%)	95675 (48%)
	Post-secondary or tertiary	96158 (54%)	97027 (49%)	68767 (38%)	68535 (34%)
Parent’s mental disorder history, *N* (%)					
	Mental disorder prior to follow-up	20868 (12%)	24374 (12%)	16693 (9.3%)	19146 (9.6%)
	No prior mental disorders	157022 (88%)	173690 (88%)	162552 (91%)	180664 (90%)
Child’s age at substance use disorder diagnosis		15 (14, 16)		15 (14, 16)	
Child’s age at psychotic disorder diagnosis		14 (12, 16)		14 (12, 16)	
Child’s age at mood disorder diagnosis		14 (13, 15)		14 (12, 15)	
Child’s age at anxiety disorder diagnosis		13 (10, 15)		13 (10, 15)	
Child’s age at eating disorder diagnosis		14 (12, 15)		14 (12, 15)	
Child’s age at intellectual disability diagnosis			5 (3, 6)		5 (3, 6)
Child’s age at developmental disorder diagnosis			7 (4, 9)		7 (4, 9)
Child’s age at childhood onset disorder diagnosis			5 (4, 7)		5 (4, 7)

**Table 2.** Descriptive statistics of the study populations

		Women	Men
Psychiatric sickness absence prior to follow-up			
	2001−2012 cohort	16891	11604
	2005−2016 cohort	16512	8984
Persons at risk at start of follow-up			
	2001−2012 cohort	177890	179245
	2005−2016 cohort	198064	199810
New cases during follow-up			
	2001−2012 cohort	30896	13690
	2005−2016 cohort	30828	16221
Emigrated during follow-up			
	2001−2012 cohort	2224	2304
	2005−2016 cohort	4202	3638
Died during follow-up			
	2001−2012 cohort	776	2032
	2005−2016 cohort	580	1607
Turned 68 during follow-up			
	2001−2012 cohort	NA^a^	346
	2005−2016 cohort	NA^a^	133
Exposed during follow-up			
	Substance use disorders	1927	2148
	Psychotic disorders	402	471
	Mood disorders	7321	8587
	Anxiety disorders	13664	15649
	Eating disorders	1828	2009
	Intellectual disabilities	2216	2258
	Developmental disorders	3635	3961
	Childhood onset disorders	42767	45583

a Less than three people were included in the cell.

**Figure 1.**
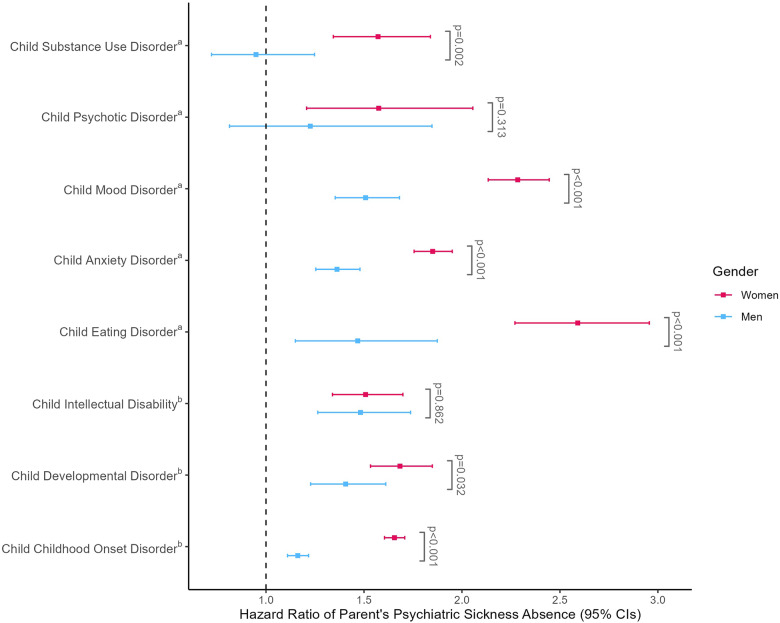
Hazard ratios and 95% confidence intervals of parents’ psychiatric sickness absence after child’s psychiatric diagnosis.

*Note: P*-values refer to the significance of the gender-exposure interaction.^a^Analyses were conducted using the 2001–2012 birth cohort.^b^Analyses were conducted using the 2005–2016 birth cohort.

Figure 1 depicts the hazard ratios for the entire follow-up period. We found an association between child’s diagnosis and parent’s sickness absence across all conditions except for men whose children were diagnosed with psychotic, or substance use disorders. The association was the greatest in women after their child had been diagnosed with an eating disorder (HR: 2.59; 95% CI: 2.27–2.96) and in men after their child was diagnosed with a mood disorder (HR: 1.51; 95% CI: 1.35–1.68). The hazard ratios were larger in women compared to men when a child was diagnosed with any of the disorders except psychotic disorders and intellectual disabilities. For exact estimates, see Supplemental Table S3.

**Figure 2.**
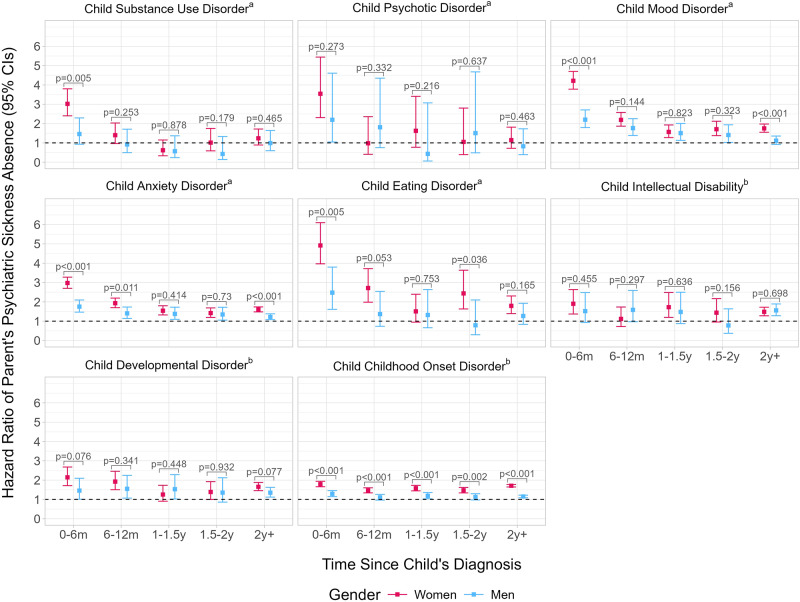
Hazard ratios and 95% confidence intervals of parents’ psychiatric sickness absence after child’s psychiatric diagnosis according to time since the child’s diagnosis.

*Note: P*-values refer to the significance of the gender-exposure interaction.^a^Analyses were conducted using the 2001–2012 birth cohort.^b^Analyses were conducted using the 2005–2016 birth cohort.

Figure 2 shows the time-dependent hazard ratios. For women especially, the HRs seemed to differ temporally with the strongest associations observed in the six months following most diagnoses. The greatest HRs were all found in women, specifically within 6 months of their child’s eating (HR: 4.92; 95% CI: 3.97–6.10) mood (HR: 4.21; 95% CI: 3.78–4.70), or psychotic disorder diagnosis (HR: 3.54; 95% CI: 2.31–5.44). The highest estimate for men was seen in the 6 months following a child’s eating disorder diagnosis (HR: 2.48; 95% CI: 1.61–3.80). There were no significant associations for men whose children were diagnosed with substance use disorders. However, unlike analyses where time was examined as a whole, there was an association in men whose children were diagnosed with a psychotic disorder in the past 6 months (HR: 2.20; 95% CI: 1.05–4.61). For women whose child was diagnosed with a mood, anxiety or childhood onset disorder, associations were found across all time periods whereas for men a similar consistent pattern was found only for anxiety disorders. Women’s hazard ratios were greater than men’s at all time points following a child’s childhood onset disorder diagnosis and during the first 6 months following a child’s substance use, mood, anxiety and eating disorder diagnosis. Additionally, there were intermittent gender differences, for example, two or more years after a child’s mood and anxiety diagnosis and consistently no evidence of gender differences at any time period following a child’s diagnosis of a psychotic disorder, intellectual disability or developmental disorder. For exact estimates, see Supplemental Table S3. For the number of new short and long sickness absences, see Supplemental Table S4. The results of analyses with short and long sickness absences can be seen in Supplemental Figures S1 and S2. Overall, while there were some differences in the point estimates across the two outcomes, the outcomes seemed to share very similar associations with the child’s diagnosis.

## Discussion

In this large nationwide register study, we found that parents of children with mental disorders or neurodevelopmental conditions had a greater risk of receiving a first psychiatric sickness absence compared to those whose children were undiagnosed, especially in the 6 months following the child’s diagnosis. Estimates differed temporally and by gender with the greatest hazard ratios observed in women shortly after a child’s eating disorder diagnosis. Findings persisted despite adjustment for potential confounders such as psychiatric co-morbidities, and parents’ educational attainment and mental disorder history. The exception to this were men whose children were diagnosed with substance use disorders for whom there was no evidence of an association with sickness absences.

To our knowledge, the present study is the most comprehensive investigation on the topic to date. Our findings indicate that parents of children with psychiatric diagnoses are at a particular risk of first-time psychiatric sickness absences. This is in line with previous studies on the topic (Hauge *et al.*, 2015; McEvilly *et al.*, 2015; Mittendorfer-Rutz *et al.*, 2019) and related research on parents’ disrupted work-family balance (Brown and Clark, 2017; Sellmaier, 2019), psychiatric distress (Cham *et al.*, 2022; Phillips *et al.*, 2023) and psychiatric morbidity (Hakulinen *et al.*, 2025). These results also elucidate previous research that indicated no immediate changes in parents’ labour market outcomes following a child’s diagnosis (Komulainen *et al.*, 2025). While parents’ distress may not be reflected in annual employment and income metrics, the present study indicates this does not necessarily mean that there are no changes in parents’ normal work routine. Given these findings, on one hand, it could be argued that the current social support structure in Finland works as intended with parents receiving monetary support and time off for psychiatric distress rather than losing employment. However, similarly to other studies (Hauge *et al.*, 2015; McEvilly *et al.*, 2015; Mittendorfer-Rutz *et al.*, 2019), it is noteworthy that these parents still reached a point of incapacity to work in a Nordic social welfare state that has existing measures for supporting parents whose children have severe health conditions (Kela, 2024). Parents’ poor mental health may reflect back on the children, and, in fact, the current Finnish mental health strategy specifically highlights that appropriate mental health services should also account for the patient’s family and their need for support (Ministry of Social Affairs and Health, 2020). Consequently, it would be important to investigate if and what kind of further support could help these parents and their families.

For the first time, we also assessed differences in the associations depending on both the child’s diagnosis and time since diagnosis. Overall, the period immediately following the child’s diagnosis was associated with the greatest excess risk. From there on, however, temporal patterns differed depending on the diagnosis. For women, several disorders were associated with consistently greater risk of a sickness absence across the whole follow-up. For men, the only diagnostic category with consistent associations was children’s anxiety disorders. Markedly, while looking at time after diagnosis as a whole, men whose children had psychotic disorders showed no evidence of an association with psychiatric sickness absences. Nevertheless, in the time-dependent analyses we observed an association in the 6 months following diagnosis. After a child’s substance use disorder diagnosis, we found no evidence of an association at any individual time period for men and an association only in the 6 months following diagnosis for women. With intellectual disabilities, there was an inconsistent temporal pattern for women and a significant excess risk only at two or more years after child’s diagnosis for men. For eating disorders, men’s associations were no longer significant after 6 months following diagnosis, whereas women’s associations were persisted through most time periods. Altogether, our findings highlight the importance of examining associations in a diagnosis-specific and time-varying manner and call for further research into the reasons behind these diagnosis-specific differences in parents’ response. These results also highlight that while the most acute need for support may be in the period just following the child’s diagnosis, depending on the diagnosis parents may require further support even long after the child’s initial diagnosis.

We also found some gender differences. In addition to there being no evidence of an association for men whose children had a substance use disorder, women’s estimates were greater than men’s at some time periods following all but psychotic disorder and intellectual disability diagnoses. Previous studies too have found greater estimates for women compared to men (McEvilly *et al.*, 2015). This could be the result of women most often being the predominant carers of family members (Sharma *et al.*, 2016). On the other hand, lower estimates in men could stem from men generally underreporting and being undertreated for mental disorders (Shi *et al.*, 2021). However, gender differences in estimates were centred around the time immediately following or more than 2 years after the child’s diagnosis. This indicates that differences are not stable over time and that despite intermittent gender differences, there was no consistent evidence of dissimilarities in parents’ responses to the child’s diagnosis. These results emphasise the importance of both including men and conducting gender-specific analyses when considering the consequences of parenting a child with a mental disorder or neurodevelopmental condition.

## Strengths and limitations

The main strength of this study was the use of nationwide register data, which allowed for a large and representative sample and objective measures of sickness absences and children’s diagnoses. Moreover, we considered various diagnoses from both primary and secondary healthcare, examined both men and women, and looked at the time-varying associations between children’s diagnoses and parents’ sickness absence. However, the current findings must be interpreted in the context of the study’s limitations. First, the study design does not allow for causal interpretations. While we controlled for potential confounders, we cannot rule out the possibility of unmeasured or residual confounding and, hence, we make no assumptions that the child’s diagnosis is causally related to the parent’s psychiatric sickness absence. We also could not account for effect modification or confounding by employment or occupation at the time of the first psychiatric sickness absence as this information was not available in the registers. Second, we only considered parents exposed after the child’s diagnosis. Thus, parents may have mistakenly been considered unexposed if their children’s diagnoses were delayed or they remained undiagnosed despite presenting with symptoms (Avlund *et al.*, 2021; Marchand *et al.*, 2006; Rice *et al.*, 2017). Additionally, we cannot rule out reverse causation, where the parent’s symptoms induce the child’s diagnosis and later also reflect as the parent’s own sickness absence. We did, however, account for this within the possibilities of our data by adjusting for parents’ mental disorder history prior to the child’s diagnosis. Our data only included psychiatric sickness absences of over nine working days, meaning that parents who received sickness absences shorter than that were defined as not having the outcome of interest. However, we believe that this measure captured most of the cases where a mental disorder required a sickness absence as a form of intervention given that official Finnish guidelines recommend that, when necessary, for example, depressive disorders warrant an initial 2–4-week absence (Current Care Guidelines, 2025). Additionally, the number of events under exposure to different disorders varies greatly especially under the time-varying analyses and when dividing the outcome to short and long sickness absences. Finally, Finland is a homogeneous social welfare country with universal healthcare, which limits the generalisability of the results.

## Conclusions

Children’s mental disorders and neurodevelopmental conditions were associated with a greater risk of parents’ first-time psychiatric sickness absence especially in the 6 months following the child’s diagnosis. Differences across parent’s gender and child’s diagnoses were found with the largest associations presenting in women whose children were recently diagnosed with an eating disorder. We call for future research on whether further support for parents could mitigate these associations. Results should be replicated in countries with different social welfare systems.

## Supporting information

10.1017/S2045796025100395.sm001 Gutvilig et al. supplementary material 1 Gutvilig et al. supplementary material

10.1017/S2045796025100395.sm002 Gutvilig et al. supplementary material 2 Gutvilig et al. supplementary material

## Data Availability

Data for this study are property of Statistics Finland, the Finnish Institute of Health and Welfare and the Social Insurance Institution of Finland. The data are available from these authorities, but restrictions apply. For more, please see www.findata.fi. The programming code is available from the authors upon request.

## Supplementary material

The supplementary material for this article can be found at https://doi.org/10.1017/S2045796025100395.
