# Supplementary material for: Mental health-related sickness absences in parents of children with mental disorders or neurodevelopmental conditions
Source: Epidemiol Psychiatr Sci. 2025 Dec 19;34:e62. doi: 10.1017/S2045796025100395 (PMC12722188; doi:10.1017/S2045796025100395)
Supplement: Gutvilig et al. supplementary material 2 — Gutvilig et al. supplementary material [file S2045796025100395sup002.docx]

**Supplemental Material**

**Supplemental Methods.** Details on Data Cleaning.

**Supplemental Table 1.** Children’s Diagnostic Categories.

**Supplemental Table 2.** Reasons for Parents’ Sickness Absences.

**Supplemental Table 3.** Exact Model Estimates.

**Supplemental Table 4.** Number of New Short and Long Sickness Absences.

**Supplemental Figure 1.** Hazard Ratios and 95% Confidence Intervals of Women’s Short and Long Psychiatric Sickness Absence After Child’s Psychiatric Diagnosis According to Time Since the Child’s Diagnosis.

**Supplemental Figure 2.** Hazard Ratios and 95% Confidence Intervals of Women’s Short and Long Psychiatric Sickness Absence After Child’s Psychiatric Diagnosis According to Time Since the Child’s Diagnosis.

**Supplemental Methods. Details on Data Cleaning.**

The data cleaning process included the following steps.

**Mental Disorders and Neurodevelopmental Conditions**. Records missing the pseudonymised id, admission, or discharge date were removed. The format of the healthcare registers was unified across years and level of healthcare and the registers were combined. From primary care, we only included diagnoses resulting from face-to-face visits. After keeping only diagnoses under the ICD-10 subchapter F and corresponding ICD-8, ICD-9, and ICPC-2 diagnoses, we removed what we determined as likely mistakes in the data: Visits with discharge preceding admission, visits occurring prior to one’s birth year and month, inpatient discharges prior to 1970, outpatient discharges prior to 1998, and primary care discharges preceding 2011 were removed. Additionally, secondary healthcare admissions or discharges and primary healthcare admissions recorded after 2020 were removed. From the remaining visits, we identified the first visit associated with the diagnosis of interest and received at or over the minimum age indicated in Methodology section.

**Psychiatric Sickness Absences.** The format of the sickness absence compensation registers was unified across years and the registers were combined and duplicate records removed. We removed records dating prior to 2003. Next, we identified the first sickness absence associated with any ICD-10 subchapter F diagnosis and noted the diagnosis associated with the absence.

**Deaths.** Records of deaths across years were combined. Individuals who had a record of the year of death but not the exact date were marked as having died on the last day of the year. For individuals with multiple death records, data was cross-referenced with annually updated demographic registers and, based on that, the latest date of death was selected.

**Emigration from Finland.** Records of all moves to, from, and within Finland were combined across years. Duplicate records were removed, and the latest move was selected. Individuals whose last move was away from Finland were marked as permanently emigrated.

**Child-Parent Linkages.** Records of children’s parents were combined across years and biological parents kept. For children with more than two recorded biological parents we cross-referenced with annual pseudonymised family ids when available and included children whose parentage could be confirmed in this way.

**Education.** Education records across years were combined. As we were interested in the highest level of education rather than the latest, records were modified to reflect the highest level of education by the end of each year. Education was classified into three groups: lower secondary or less (in Finland this is indicated by no education records), upper secondary, and post-secondary. Individuals could only be missing education records if their pseudonymised id was not found in any of the education registers.

**Supplemental Table 1. Children’s Diagnostic Categories.**

| **Diagnostic Category** | **ICD-10** | **Abbreviation** | **ICPC-2 Equivalents** |
| --- | --- | --- | --- |
| Mental and behavioural disorders due to psychoactive substance use | F10-F19 | Substance use disorders | P16, P15, P17, P19 |
| Schizophrenia, schizotypal and delusional disorders | F20-F29 | Psychotic disorders | P72 |
| Mood (affective) disorders | F30-F39 | Mood disorders | P73 |
| Neurotic, stress-related and somatoform disorders | F40-F48 | Anxiety disorders | P79, P74, P02, P82, P75, P78 |
| Eating disorders | F50 | Eating disorders | P86 |
| Intellectual disability | F70-F79 | Intellectual disabilities | P85 |
| Pervasive developmental disorders | F84 | Developmental disorders | - |
| Behavioral and emotional disorders with onset in childhood and adolescence | F90-F98 | Childhood onset disorders | P81, P22, P23, P10, P12, P13, P11 |

ICD-10 = International Statistical Classification of Diseases and Related Health Problems 10^th^ Revision. ICPC-2 = International Classification of Primary Care 2^nd^ Edition.

**Supplemental Table 2. Reasons for Parents’ Sickness Absences.**

|  | **Women** | | **Men** | |
| --- | --- | --- | --- | --- |
|  | 2001-2012 Cohort | 2005-2016 Cohort | 2001-2012 Cohort | 2005-2016 Cohort |
| Parent's Diagnosis |  |  |  |  |
| Psychotic disorders (F20-F29) | 412 (1.3%) | 437 (1.4%) | 366 (2.7%) | 486 (3.0%) |
| Depressive disorders (F32-F33, F39) | 12747 (41%) | 12863 (42%) | 6029 (44%) | 6923 (43%) |
| Other mood disorders (F30-F31, F34) | 444 (1.4%) | 505 (1.6%) | 308 (2.2%) | 386 (2.4%) |
| Anxiety disorders (F40-F48) | 14087 (46%) | 13874 (45%) | 5396 (39%) | 6659 (41%) |
| Sleep disorders (F51) | 2973 (9.6%) | 2873 (9.3%) | 1215 (8.9%) | 1333 (8.2%) |
| Other psychiatric disorders^a^ | 223 (0.7%) | 255 (0.8%) | 371 (2.7%) | 428 (2.6%) |
| Multiple psychiatric disorders | 10 (<0.1%) | 21 (<0.1%) | 5 (<0.1%) | 6 (<0.1%) |

^a^Other psychiatric disorders include any other ICD-10 subchapter F diagnoses.

**Supplemental Table 3. Exact Model Estimates**

|  |  | **Women** | **Men** | **Interaction** | |
| --- | --- | --- | --- | --- | --- |
|  |  | HR (95% CIs) | HR (95% CIs) | HR (95% CIs)^a^ | P-value |
| Child's Substance Use Disorder | |  |  |  |  |
|  | whole follow-up | 1.57 (1.34-1.84) | 0.95 (0.72-1.25) | 1.66 (1.21-2.27) | 0.002 |
|  | 0-6 months after exposure | 3.02 (2.4-3.8) | 1.46 (0.93-2.29) | 2.07 (1.25-3.43) | 0.005 |
|  | 6-12 months after exposure | 1.4 (0.97-2.03) | 0.92 (0.49-1.71) | 1.52 (0.74-3.14) | 0.253 |
|  | 1-1.5 years after exposure | 0.62 (0.33-1.15) | 0.57 (0.24-1.37) | 1.09 (0.37-3.18) | 0.878 |
|  | 1.5-2 years after exposure | 1.01 (0.59-1.75) | 0.43 (0.14-1.33) | 2.36 (0.67-8.3) | 0.179 |
|  | more than 2 years after exposure | 1.24 (0.89-1.72) | 0.99 (0.6-1.64) | 1.25 (0.69-2.29) | 0.465 |
| Child's Psychotic Disorder | |  |  |  |  |
|  | whole follow-up | 1.58 (1.21-2.06) | 1.23 (0.81-1.85) | 1.28 (0.79-2.09) | 0.313 |
|  | 0-6 months after exposure | 3.54 (2.31-5.44) | 2.2 (1.05-4.61) | 1.61 (0.69-3.8) | 0.273 |
|  | 6-12 months after exposure | 0.98 (0.41-2.36) | 1.81 (0.75-4.35) | 0.54 (0.16-1.87) | 0.332 |
|  | 1-1.5 years after exposure | 1.62 (0.77-3.41) | 0.43 (0.06-3.07) | 3.76 (0.46-30.53) | 0.216 |
|  | 1.5-2 years after exposure | 1.05 (0.39-2.8) | 1.51 (0.49-4.68) | 0.7 (0.16-3.12) | 0.637 |
|  | more than 2 years after exposure | 1.14 (0.72-1.81) | 0.82 (0.39-1.73) | 1.39 (0.58-3.32) | 0.463 |
| Child's Mood Disorder | |  |  |  |  |
|  | whole follow-up | 2.28 (2.13-2.45) | 1.51 (1.35-1.68) | 1.51 (1.34-1.72) | < 0.001 |
|  | 0-6 months after exposure | 4.21 (3.78-4.7) | 2.2 (1.79-2.71) | 1.91 (1.52-2.41) | < 0.001 |
|  | 6-12 months after exposure | 2.19 (1.87-2.58) | 1.76 (1.38-2.25) | 1.24 (0.93-1.66) | 0.144 |
|  | 1-1.5 years after exposure | 1.57 (1.27-1.93) | 1.51 (1.13-2.01) | 1.04 (0.73-1.48) | 0.823 |
|  | 1.5-2 years after exposure | 1.71 (1.38-2.12) | 1.41 (1.02-1.94) | 1.21 (0.83-1.78) | 0.323 |
|  | more than 2 years after exposure | 1.75 (1.55-1.98) | 1.12 (0.92-1.35) | 1.57 (1.25-1.97) | < 0.001 |
| Child's Anxiety Disorder | |  |  |  |  |
|  | whole follow-up | 1.85 (1.76-1.95) | 1.36 (1.25-1.48) | 1.36 (1.23-1.49) | < 0.001 |
|  | 0-6 months after exposure | 2.98 (2.7-3.28) | 1.75 (1.47-2.09) | 1.7 (1.39-2.08) | < 0.001 |
|  | 6-12 months after exposure | 1.93 (1.7-2.19) | 1.4 (1.14-1.73) | 1.38 (1.08-1.76) | 0.011 |
|  | 1-1.5 years after exposure | 1.54 (1.32-1.8) | 1.37 (1.09-1.73) | 1.12 (0.85-1.47) | 0.414 |
|  | 1.5-2 years after exposure | 1.42 (1.19-1.69) | 1.35 (1.05-1.72) | 1.05 (0.78-1.42) | 0.73 |
|  | more than 2 years after exposure | 1.6 (1.48-1.74) | 1.22 (1.08-1.38) | 1.32 (1.14-1.52) | < 0.001 |
| Child's Eating Disorder | |  |  |  |  |
|  | whole follow-up | 2.59 (2.27-2.96) | 1.47 (1.15-1.87) | 1.76 (1.34-2.33) | < 0.001 |
|  | 0-6 months after exposure | 4.92 (3.97-6.1) | 2.48 (1.61-3.8) | 1.99 (1.23-3.21) | 0.005 |
|  | 6-12 months after exposure | 2.71 (1.98-3.72) | 1.37 (0.74-2.54) | 1.99 (0.99-3.98) | 0.053 |
|  | 1-1.5 years after exposure | 1.51 (0.95-2.39) | 1.32 (0.66-2.64) | 1.14 (0.5-2.63) | 0.753 |
|  | 1.5-2 years after exposure | 2.44 (1.63-3.64) | 0.79 (0.3-2.1) | 3.09 (1.07-8.92) | 0.036 |
|  | more than 2 years after exposure | 1.79 (1.39-2.3) | 1.27 (0.83-1.93) | 1.41 (0.87-2.3) | 0.165 |
| Child's Intellectual Disability | |  |  |  |  |
|  | whole follow-up | 1.51 (1.34-1.7) | 1.48 (1.26-1.74) | 1.02 (0.84-1.24) | 0.862 |
|  | 0-6 months after exposure | 1.9 (1.37-2.64) | 1.52 (0.93-2.48) | 1.25 (0.69-2.26) | 0.455 |
|  | 6-12 months after exposure | 1.12 (0.72-1.73) | 1.58 (0.97-2.59) | 0.7 (0.37-1.36) | 0.297 |
|  | 1-1.5 years after exposure | 1.72 (1.2-2.48) | 1.48 (0.87-2.5) | 1.17 (0.62-2.21) | 0.636 |
|  | 1.5-2 years after exposure | 1.44 (0.96-2.17) | 0.78 (0.37-1.64) | 1.85 (0.79-4.3) | 0.156 |
|  | more than 2 years after exposure | 1.48 (1.28-1.72) | 1.56 (1.28-1.89) | 0.95 (0.75-1.22) | 0.698 |
| Child's Developmental Disorder | |  |  |  |  |
|  | whole follow-up | 1.68 (1.53-1.85) | 1.41 (1.23-1.61) | 1.2 (1.02-1.41) | 0.032 |
|  | 0-6 months after exposure | 2.14 (1.72-2.68) | 1.46 (1.01-2.1) | 1.47 (0.96-2.26) | 0.076 |
|  | 6-12 months after exposure | 1.92 (1.5-2.46) | 1.55 (1.07-2.25) | 1.24 (0.8-1.94) | 0.341 |
|  | 1-1.5 years after exposure | 1.25 (0.91-1.73) | 1.53 (1.02-2.28) | 0.82 (0.49-1.37) | 0.448 |
|  | 1.5-2 years after exposure | 1.39 (1-1.92) | 1.35 (0.86-2.12) | 1.02 (0.59-1.79) | 0.932 |
|  | more than 2 years after exposure | 1.65 (1.45-1.88) | 1.35 (1.12-1.63) | 1.23 (0.98-1.54) | 0.077 |
| Child's Childhood Onset Disorder | |  |  |  |  |
|  | whole follow-up | 1.66 (1.6-1.71) | 1.16 (1.11-1.22) | 1.42 (1.35-1.5) | < 0.001 |
|  | 0-6 months after exposure | 1.79 (1.66-1.94) | 1.29 (1.14-1.46) | 1.39 (1.2-1.6) | < 0.001 |
|  | 6-12 months after exposure | 1.46 (1.34-1.6) | 1.1 (0.97-1.26) | 1.33 (1.13-1.56) | < 0.001 |
|  | 1-1.5 years after exposure | 1.58 (1.44-1.73) | 1.19 (1.04-1.36) | 1.33 (1.13-1.56) | < 0.001 |
|  | 1.5-2 years after exposure | 1.47 (1.34-1.62) | 1.12 (0.97-1.29) | 1.31 (1.1-1.56) | 0.002 |
|  | more than 2 years after exposure | 1.71 (1.64-1.78) | 1.15 (1.09-1.22) | 1.48 (1.39-1.59) | < 0.001 |

HR = Hazard ratio, CI = Confidence interval. ^a^ For gender estimates, man is the reference category.

**Supplemental Table S3. Number of New Short and Long Sickness Absences.**

| **New Cases** | **Women** | **Men** |
| --- | --- | --- |
| Short Absences | |  |
| 2001–2012 Cohort | 22336 | 9443 |
| 2005–2016 Cohort | 21702 | 11459 |
| Long Absences | |  |
| 2001–2012 Cohort | 8560 | 4247 |
| 2005–2016 Cohort | 9126 | 4762 |

**Supplemental Figure 1. Hazard Ratios and 95% Confidence Intervals of Women’s Short and Long Psychiatric Sickness Absence After Child’s Psychiatric Diagnosis According to Time Since the Child’s Diagnosis.**


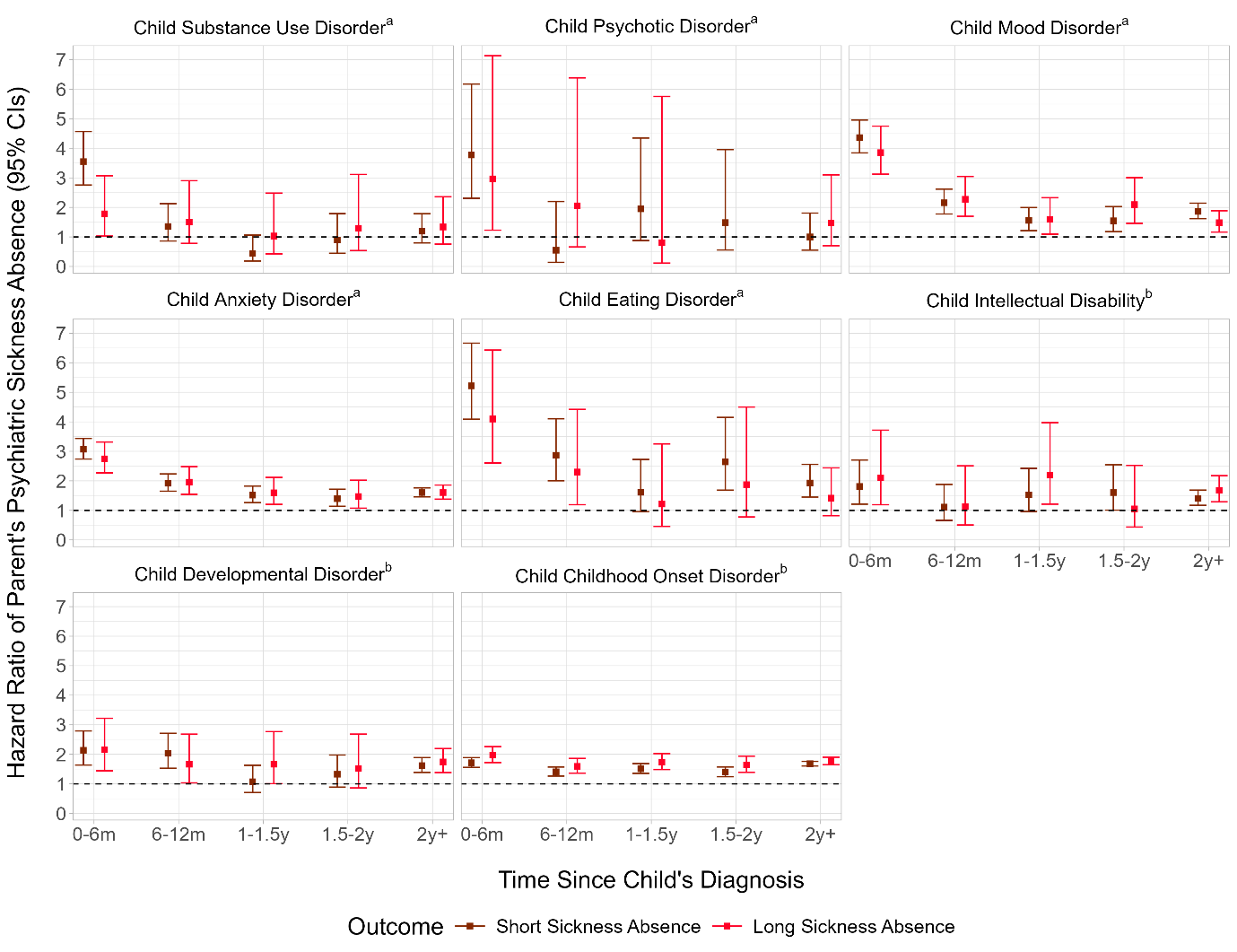


Note: Hazard ratios and confidence intervals are not shown when there are no events in the exposure group as hazard ratios calculated with an event ratio of 0/x are numerically infinite.

^a^ Analyses were conducted using the 2001–2012 birth cohort

^b^ Analyses were conducted using the 2005–2016 birth cohort

**Supplemental Figure 2. Hazard Ratios and 95% Confidence Intervals of Men’s Short and Long Psychiatric Sickness Absence After Child’s Psychiatric Diagnosis According to Time Since the Child’s Diagnosis.**
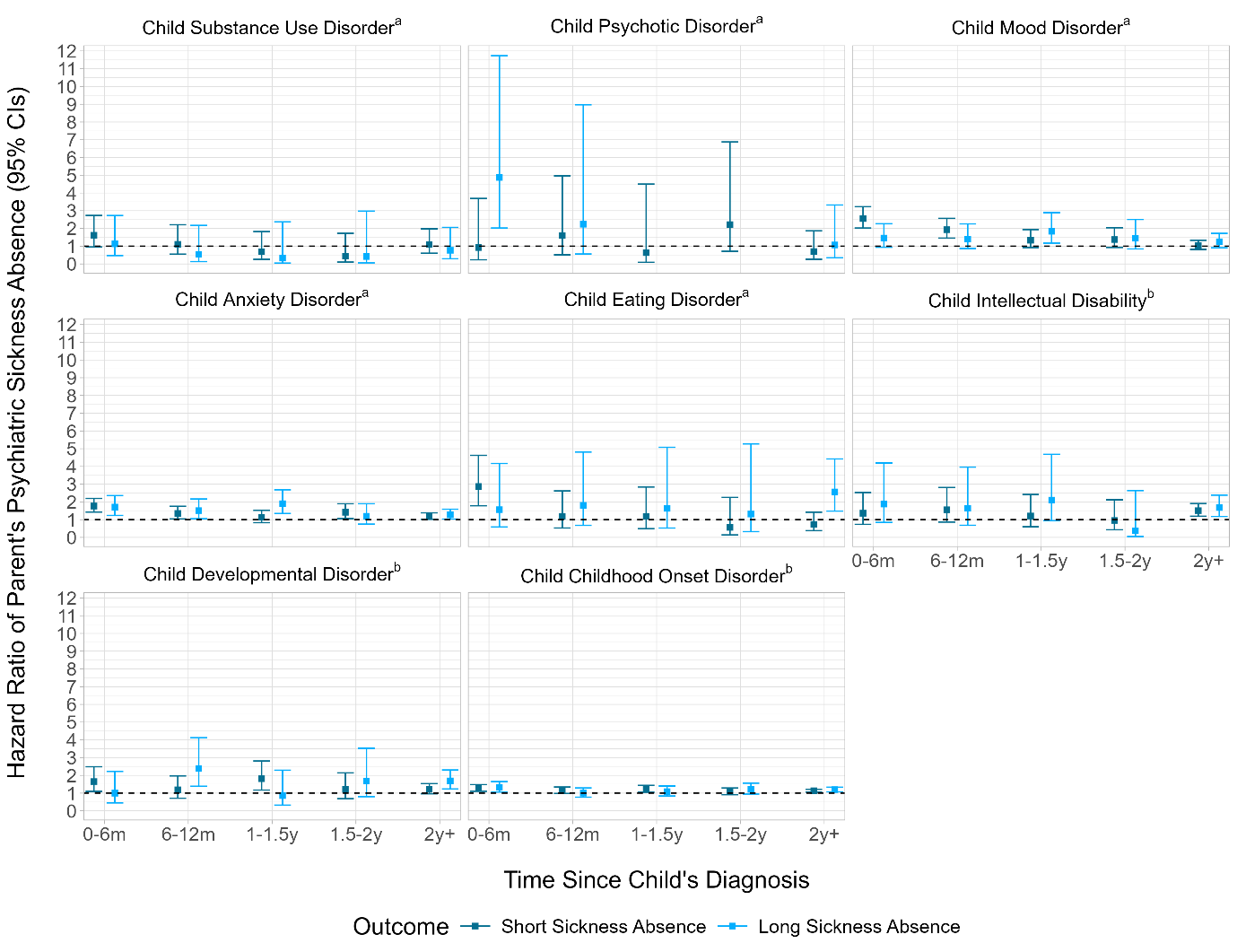


Note: Hazard ratios and confidence intervals are not shown when there are no events in the exposure group as to hazard ratios calculated with an event ratio of 0/x are numerically infinite.

^a^ Analyses were conducted using the 2001–2012 birth cohort

^b^ Analyses were conducted using the 2005–2016 birth cohort
